# De‐escalation or discontinuation of tyrosine kinase inhibitor in patients with chronic myeloid leukemia: A multicentral, open‐label, prospective trial in China

**DOI:** 10.1002/jha2.550

**Published:** 2022-09-19

**Authors:** Jie Luo, Xin Du, Jin Lou, Jianwei Wu, Liping Ma, Jixian Huang, Liangtuo Wang, Chuanqing Tu, Zelin Liu, Liya Chen, Yaxian Tan, Dongmei Luo, Hanyin Liang, Changxin Yin, Rui Cao, Xuan Zhou, Qifa Liu, Xiaoli Liu, Na Xu

**Affiliations:** ^1^ Department of Hematology Nanfang Hospital Southern Medical University Guangzhou China; ^2^ Department of Hematology, Shenzhen Second People's Hospital, The First Affiliated Hospital of Shenzhen University Shenzhen University School of Medicine Shenzhen China; ^3^ Department of Hematology Jinan University Affiliated Jiangmen Hospital of Traditional Chinese Medicine Jiangmen Guangdong China; ^4^ Department of Hematology, Sun Yat‐sen Memorial Hospital Sun Yat‐sen University Guangzhou China; ^5^ Department of Hematology, Yuebei People's Hospital Shantou University Shaoguan Guangdong China; ^6^ Department of Hematology People's hospital of Yang Jiang Yang Jiang Guangdong China; ^7^ Department of Hematology, Bao' an District People Hospital The Second Affiliated Hospital of Shenzhen University Shenzhen China; ^8^ Department of Hematology Huazhong University of Science and Technology Union Shenzhen Hospital (Nanshan Hospital) Shenzhen China; ^9^ Department of Medical Quality Management Nanfang Hospital Southern Medical University Guangzhou China

**Keywords:** chronic myeloid leukemia, de‐escalation TKI, treatment‐free remission

## Abstract

**Background**: Long‐term treatment‐free remission (TFR) represents a new goal for chronic myeloid leukemia (CML). Optimizing dose of tyrosine kinase inhibitors (TKIs) in the CML treatment maybe a new challenge to maintain effective and improving patients’ quality of life. We hypothesized that administration of low‐dose TKIs does not compromise major molecular response (MMR) in patients with CML who have a deep molecular response (DMR).

**Methods**: We did an open‐label, randomized trial at eight hospitals in China. Eligible CML‐CP patients (aged 18–70 years) had shown continuous response to TKI more than 5 years and maintained MR4.5 (BCR‐ABLIS ≤ 0.0032%) in recent 18 months. Patients were randomly assigned (1:1) to the TKI de‐escalation group or the discontinuation group. Randomization was done with permuted blocks (block size four) and implemented through an interactive web‐based randomization system. Recurrence was defined as the single sample with real time Quantitative PCR (RT‐qPCR) measurement greater than 0.1% (MMR). The primary endpoint was 12‐month MMR rate in patients who received de‐escalation or discontinuation of TKIs. This study was registered at ClinicalTrials.gov (NCT04143087).

**Results**: Around 125 patients were enrolled between October 23, 2019 and October 31, 2020, 62 patients received dose de‐escalation of TKIs, while 63 patients in the discontinuation group. In the de‐escalation group, molecular recurrence‐free survival at 12 months was 88.32% (95% CI 79%–98%), whereas molecular recurrence‐free survival in the discontinuation group at 12 months was 59.98% (95% CI 47–73). No progressions occurred at the data cut‐off date. All 29 recurrence cases restart TKI treatment returned to MMR. Cytolytic NK cells as a proportion of lymphocyte cells were significantly increased from baseline after 6 months whether in the de‐escalation or TKIs cessation group (*P* = 0.048, 0.001, respectively); compared with the relapsing patients, Tregs proportion was decreased (*P* = 0.003), and higher proportion of NK cells were found in non‐relapsing patients whether in TKI de‐escalation or discontinuation group (*P* = 0.011, 0.007, respectively). We also found that the de‐escalation group showed better disease‐specific HRQOL in regards to its impact on emotional functioning, fatigue, pain, and financial difficulties.

**Conclusion**: With 88.32% MMR in 12‐months follow‐up after de‐escalation TKIs’ treatment, dose‐halving could become a new treatment paradigm for CML patients who with DMR under continuing maintenance therapy with TKIs.

## 1 INTRODUCTION

Tyrosine kinase inhibitors (TKIs) had dramatically improved the treatment and prognosis of chronic myeloid leukemia (CML) patients. The life expectancy of most CML patients is similar to healthy age matched individuals [1, 2]. A new goal for treating CML is survival at good quality of life, with treatment discontinuation in sustained deep molecular response (DMR) and treatment‐free remission (TFR). Unexpectedly, the benefit of TFR policy is restricted to no more than 15%–25% of all CML patient population [3, 4, 5]. Meaning the majority of patients are at risk of lifelong exposure to TKIs, long‐term treatment with TKIs is accompanied with high cost of TKIs and adverse events (AEs), which negatively impact patients’ quality of life and may even cause significant morbidity and mortality [6].

TKIs’ dose de‐escalation is considered as a way of dealing with such problems. Recently, retrospective analyses of large‐cohort clinical trials have shown that TKIs dose reduction did not compromise efficacy and improved patients’ quality of life [5, 7]. Furthermore, the DESTINY trial showed that de‐escalation of TKIs may improve TFR than discontinuation [8, 9, 10]. Fassoni et al. [11] used a mathematical model to verify that dose‐halving TKI does not lead to a reduction of long‐term treatment efficiency patients who have already achieved sustained remission. While there was substantial data on efficacy and safety of TKI dose reduction, but there was lack of the underlying mechanism and health‐related quality of life (HRQOL) data of patients with TKIs’ dose reduction.

Immunologic surveillance of residual leukemic cells (LC) is hypothesized to be one of the critical factors in successful TFR [5, 12]. DMR is associated with increased NK and CD8+ T‐cell numbers, and decreased Tregs in the peripheral blood of CML patients. Likewise, successful TFR has been linked to increased NK/CD8+ T‐cells and decreased Tregs [5, 13‐14]. However, it is an ambiguous inference of individual immunologic configurations based on TKI dose reduction.

All TKIs have potential immunosuppressive effects.

Therefore, we performed this multicenter study to explore impact of dose de‐escalation or discontinuation on Chinese CML patients, aiming to provide some evidence about optimizing dose of TKIs.

## 2 METHODS

### 2.1 Study design and participants

This trial was conducted at eight hospitals in China. The inclusion criteria include: age ≥18 years; in first chronic phase with a known BCR‐ABL1 transcript (any transcript type was permitted); ≥5 years of therapy with full dose of TKIs’ therapy (either imatinib, nilotinib, or dasatinib); had monitored the BCR‐ABL1 transcripts frequently through real time Quantitative PCR (RT‐qPCR) analysis in recent 18 months, each with 32,000 or more ABL1 control transcripts and had persistent BCR‐ABL1^IS^≤0.0032% (also known as MR4.5, defined as a 4.5‐log reduction in the BCR‐ABL1 transcript according to the international scale). Patients at the accelerated phase (AP)/blast crisis (BC) of CML, or combined with mutations in the ABL kinase region, patients who received allogeneic hematopoietic stem‐cell transplantation, or were treated with any other immunotherapy except interferon, and pregnancy or breastfeeding, were excluded from this study.

All individual entrants provided written informed consent before enrollment, and the trial was conducted in line with the principles of the Declaration of Helsinki. This study was registered at ClinicalTrials.gov (NCT04143087).

### 2.2 TKIs dose procedure

Eligible patients were randomly allocated to TKIs’ de‐escalation group or discontinuation group. In the de‐escalation group, participants dose‐halving TKIs: imatinib 200 mg once daily, dasatinib 50 mg once daily, or nilotinib 300 mg or 400 mg once daily. RT‐qPCR analyses were done monthly for the first 6 months, every 2 months for the subsequent 6 months, and we expressed all BCR‐ABL1 ratios according to the international scale. Molecular recurrence was defined as loss of major molecular response (MMR; BCR‐ABL1^IS^ > 0.1%) at a single timepoint. In these cases, all patients were required to readministered TKIs with entry dose, and we continued monitoring monthly until MMR was reached again.

Lymphocyte subsets were examined by flow cytometry at trial entry, after 6 months of half‐dose therapy and treatment cessation, and when patients molecular relapse. The lymphocyte fraction was examined by flow cytometry with FACSCalibur cytometer and BD Cell Quest software, version 3.3 (Becton Dickinson, Franklin Lakes, NJ, USA). All antibodies were purchased from Becton Dickinson. The lymphocyte subsets were defined as follows: CD3^+^ T cells, CD4 T cells (CD3^+^CD4^+^), CD8 T cells (CD3^+^CD8^+^), NK cells (CD3^–^CD56^+^, CD3^–^CD56^+^CD16^+^ and CD3^–^CD56^+^CD16^–^), T regulatory cells (CD3^+^CD4^+^CD25^high^CD127^–^Foxp3^+^), B cells (CD19^+^).

HRQOL was accessed with the EORTC QLQ‐C30 questionnaire in 6 months after patients’ enrollment [15], which was composed of 15 territories and 30‐item measures, including five functional scales: physical, role, cognitive, emotional, and social functioning; three symptom scales: fatigue, pain, nausea, and vomiting; six single items and a global QOL scale. Except the global QOL scale was rated using a positive score range 1–7, the other parameters were rated using a four‐point score (1 = not at all,2 = a little bit,3 = quite a bit,4 = very much). In order to make the scores in each domain comparable, we converted the raw scores into standard scores with a value of 0–100. Notably, higher scores on functional and global QOL scales indicate better functioning, whereas higher scores on symptom scales indicate greater symptom burden.

### 2.3 Outcomes

The primary end‐point of this study was the MMR rate after a follow‐up of 12 months in patients who received de‐escalation or discontinuation of TKIs. The secondary endpoints include: proportion of relapsed patients who regained molecular remission after TKIs’ re‐challenge, and time to MMR recovery (defined as the time from the date of confirmed loss of MMR to the date of MMR recovery) and survival; and the immunologic configurations based on treatment alterations; the difference of HRQOL between the de‐escalation and the discontinuation group.

### 2.4 Statistical analysis

The sample size was required to provide the study with a significance level of 0.05 and a power of 90%; calculated assuming a worst‐case scenario (TKI stop proportion of relapsing patients = 0.5; TKI de‐escalation proportion of relapsing patients = 0.8; group allocation of 1:1 in either direction; 11% dropouts), and estimated that a minimum of total planned sample size was 117 patients (58 in one group, 59 in another group).

We structured the trial as two cohorts, de‐escalation group and discontinuation group. Statistical analyses were done with IBM‐SPSS software package, version 22. No adjustment for multiple testing or missing data was incorporated. Continuous variables were summarized using median and ranges, categorical variables were expressed as frequencies and proportions. Descriptive analyses comparing cohorts were performed using the chi‐square test, Wilcoxon matched‐pairs signed ranked test, or Mann–Whitney *U*‐test. The molecular relapse‐free survival was estimated using the Kaplan–Meier method and reported with 95% confidence interval (CI), comparison of survival curves was performed using the log‐rank test through GraphPad Prism v7(GraphPad Software Inc, La Jolla, CA, USA). For the estimation of time to MMR recovery, cumulative incidence analysis was used. Univariate and multivariate Cox regression analyses were used to select the factors impact on successful recurrence‐free survival from various trial entry characteristics. *p* < 0.05 was considered significant.

## 3 RESULTS

### 3.1 Patient characteristics

**FIGURE 1:**
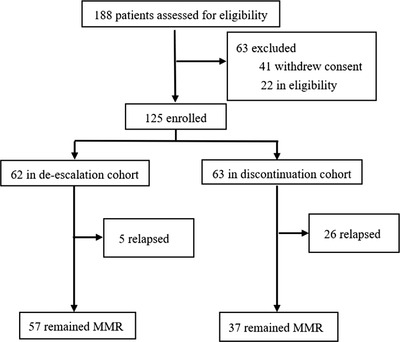
Trial profile

The trial was conducted between October 23, 2019 and October 31, 2020 at eight centers in South China, 188 patients were screened and 63 (33.51%) were excluded (Figure 1). These were composed of 41 patients who withdrew due to concern over additional visits and RT‐qPCR analyses (requested monthly for the first 6 months, every 2 months for the subsequent 6 months), and 22 who were ineligible (18 combined with mutations in the ABL kinase region, 4 pregnancy).

A total of 125 patients enrolled in this study, 62 patients enrolled into the de‐escalation group and 63 patients enter into the discontinuation group (Figure 1). Baseline demographic and clinical characteristics were summarized in Table 1. The median duration of TKI therapy was 72 months (range 60–168 months) in the de‐escalation cohort and 79 months (range 60–180 months) in the discontinuation cohort, whereas the median duration of MR^4.5^ was 36 months (range 18–156 months) and 40 months (range 18–124 months), respectively. Although these differences in baseline characteristics were not significant.

**TABLE 1:** Baseline demographic and clinical characteristics of the 125 patients enrolled in this trial

	**TKI de‐escalation (*n* = 62)**	**TKI discontinuation (*n* = 63)**	**Overall (*n* = 125)**	***P***
Median age (years)	49 (18–76)	47 (18–82)	48 (18–82)	0.061
Sex				0.328
Male	29 (47%)	35 (56%)	64 (51%)	
Female	33 (53%)	28 (44%)	61 (49%)	
Sokal risk				0.816
Low	33 (53%)	35 (56%)	68 (54%)	
Intermediate	22 (35%)	21 (33%)	43 (34%)	
High	7 (11%)	7 (11%)	14 (11%)	
Previous IFN therapy				0.979
Yes	5 (8%)	5 (8%)	10 (8%)	
No	57 (92%)	58 (92%)	115 (92%)	
Medication				0.585
Imatinib	48 (77%)	52 (82%)	100 (80%)	
Nilotinib	6 (10%)	8 (13%)	14 (11%)	
Dasatinib	8 (13%)	3 (5%)	11 (8%)	
First‐line treatment	57 (92%)	60 (95%)	117 (94%)	0.452
Second‐line treatment				
Resistance	3 (5%)	2 (3%)	5 (4%)	
Intolerance	2 (3%)	1 (2%)	3 (2%)	
Median duration of TKIs (months)	72 (60–168)	79 (60–180)	72 (60–180)	0.152
Median duration in MR^4.5^(months)	36 (18–156)	40 (18–124)	36 (18–156)	0.052

*Note*: There was no significant difference in baseline demographic and clinical characteristics between TKI de‐escalation and discontinuation groups. TKIs, tyrosine kinase inhibitors; MR^4.5^, deep molecular response (BCR‐ABL^IS^ ≤ 0.0032%).

### 3.2 **Molecular relapses and molecular** r**elapse‐free survival**

**FIGURE 2:**
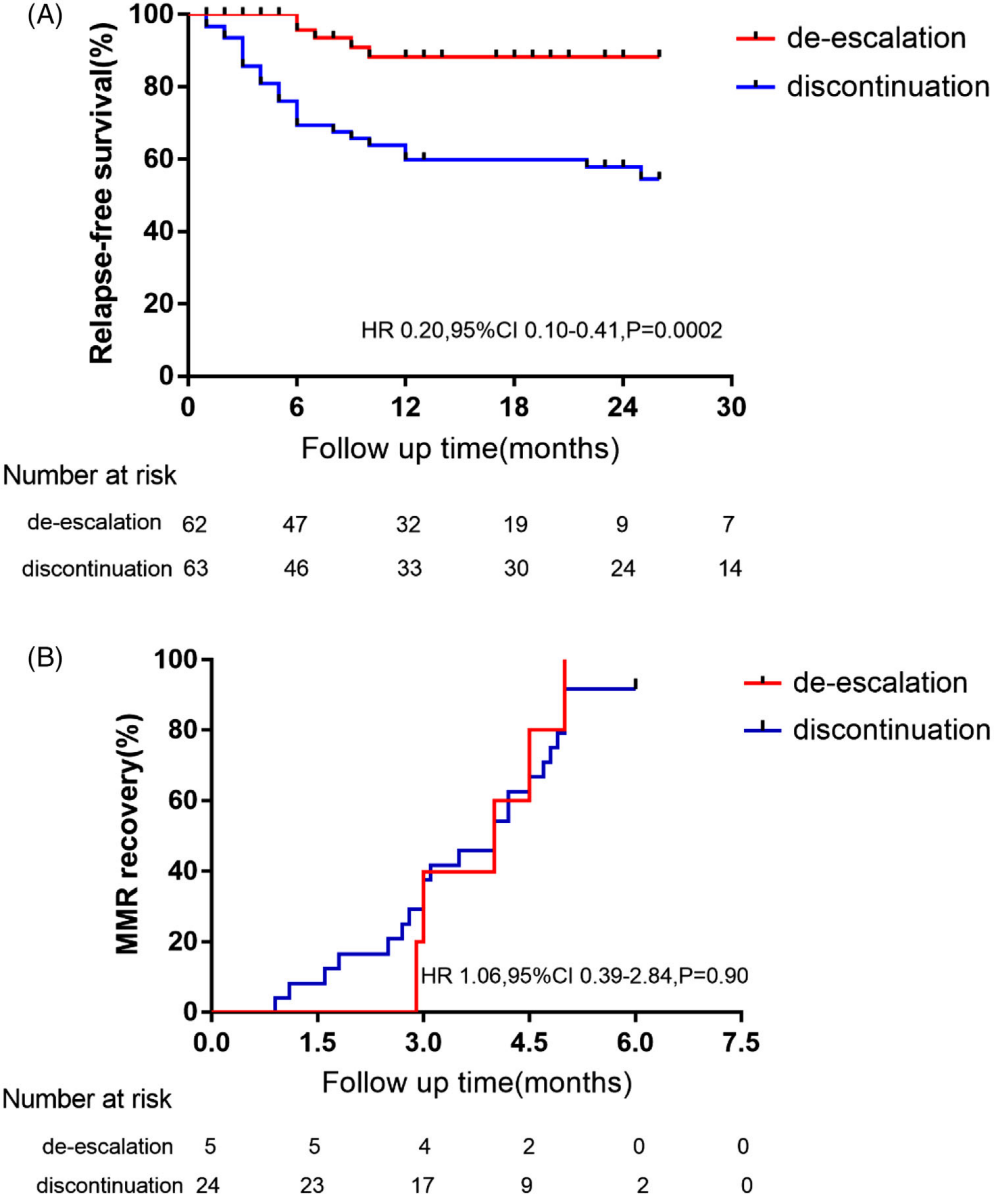
Molecular recurrence‐free survival after tyrosine kinase inhibitor (TKI) de‐escalation or discontinuation (A) and time to MMR recovery after TKI reintroduction (B) HR, hazard ratio; 95% CI, 95% confidence interval; MMR, major molecular response (BCR‐ABL^IS^ < 0.1%). For each survival plot, a corresponding log‐rank HR, 95% CI and *P*‐value were shown

During the 12 months of half‐dose therapy, 5 patients (8.06%) had molecular recurrence (loss of MMR), whereas molecular recurrence was seen in 24 patients (38.10%) in the discontinuation group. Molecular recurrence‐free survival was significantly lower in the discontinued group [59.98% (95% CI 47–73) versus 88.32% (95% CI 79–98); *p* = 0.0002; Figure 2A)].

Patients with recurrences were required to resume the full dose of their entry TKI, and all complied with this. Of the 29 recurrences across the trial, 27 returned to MMR within 5 months of TKI resumption, with no significant difference (*p* = 0.90) between the discontinuation group and the de‐escalation group. Another two patients in the discontinuation group opted to dasatinib (their entry TKI) 50 mg once daily less than full dose , and returned to MMR within 6 months. Notably, during 12‐month follow‐up, no death cases reported and no patient underwent disease progression to AP/BC of CML.

During the 12‐month observation period, TKI‐related AEs (nausea and vomiting, diarrhea, anorexia, abdominal discomfort, and night sweat) all improved in both groups. However, we have found that there were 24%, 28.8%, and 15.2% patients complained about aggravation or new development of fatigue, musculoskeletal pain, and pruritus after TKI de‐escalation or discontinuation (Table 2). And these three worsened or newly developed symptoms were more occurred in the discontinuation group, the incidence of musculoskeletal pain shows the significant difference between the two groups (*P* = 0.021). Musculoskeletal pain was involved to the whole body particularly the upper joint, and it can be controlled by non‐steroidal anti‐inflammatory drugs (NSAID) with the median duration time of 5 months.

**TABLE 2:** Aggravation or new development symptoms after TKI de‐escalation or discontinuation (*N*, %)

**Symptoms**	**De‐escalation**	**Discontinuation**	**Total**	***P* value**
Fatigue	14 (22.58%)	16 (25.4%)	30 (24%)	0.712
Grade 1–2	13	10	23	
Grade 3–4	1	6	7	
Musculoskeletal pain	12 (19.23%)	24 (33.33%)	36 (28.8%)	0.021
Grade 1–2	11	21	32	
Grade 3–4	1	3	4	
Pruritus	7 (11.54%)	12 (19.05%)	19 (15.2%)	0.227
Grade 1–2	6	9	15	
Grade 3–4	1	3	4	

### 3.3 Predictive factors for molecular recurrence

The risk of recurrence of patients in the discontinuation group was 4.94 times than in the de‐escalation group (95% CI 1.89–12.89, *P* = 0.001; Table 3). In univariate analysis, no correlation was seen in the Sokal score and medication with recurrence in the discontinuation or the de‐escalation group. But a significant association was found among patients with continuous TKI treatment and the longer duration of MR^4.5^, and the fewer of recurrences. Patients who treated ongoing TKIs for more than 6 years had a 0.36 times greater risk of recurrence than those treated less than 6 years (95% CI 0.18–0.75, *P* = 0.006; Table 3), and maintained MR^4.5^ for less than 36 months were 3.33 times more likely to relapse than those maintained over 3 years (95% CI 0.14–0.62, *P* = 0.001; Table 3).

**TABLE 3:** Univariate and multivariable analysis of various parameters' associations with molecular recurrence

**Characteristic**	**Univariate**		**Multivariable**	
	**HR (95**% **CI)**	***P* value**	**HR (95**% **CI)**	***P* value**
Group				
De‐escalation vs. discontinuation	4.94 (1.89–12.89)	0.001*	6.47 (2.62–16.00)	<0.001*
Sokal score				
Low vs. high to intermediate	1.61 (0.79–3.29)	0.192		
Medication				
Imatinib vs. second generation	1.47 (0.63–3.41)	0.375		
Median duration of TKIs (months)				
< 72 vs. ≥72	0.36 (0.18–0.75)	0.006*	0.41 (0.19–0.89)	0.024*
Median duration in MR^4.5^ (months)				
< 36 vs. ≥36	0.30 (0.14–0.62)	0.001*	0.26 (0.14–0.57)	0.001*

*Note*: Where relevant, the HR refers to the probability of recurrence for the parameter relative to the comparable one (e.g., de‐escalation vs. discontinuation, male vs. female, Sokal score low risk vs. high to intermediate risk). HR, hazard ratio. Second generation = nilotinib and dasatinib. TKIs, tyrosine kinase inhibitors; MR^4.5^, deep molecular response (BCR‐ABL^IS^ ≤ 0.0032%). ^*^ *P*< 0.05 was considered statistically significant.

Using the backward elimination method, a *p*‐value of 0.1 acts as a threshold to enroll in the multivariate analysis. The variables included in the multivariate analysis in this trial are as follows: group, total TKI treatment time, and duration of MR^4.5^. The results showed that de‐escalation group, longer duration of TKI treatment, and the time of MR^4.5^ maintained were the protective factor in molecular remission (Table 3).

### 3.4 The effect on immune function of de‐escalating/discontinuing TKIs

**FIGURE 3:**
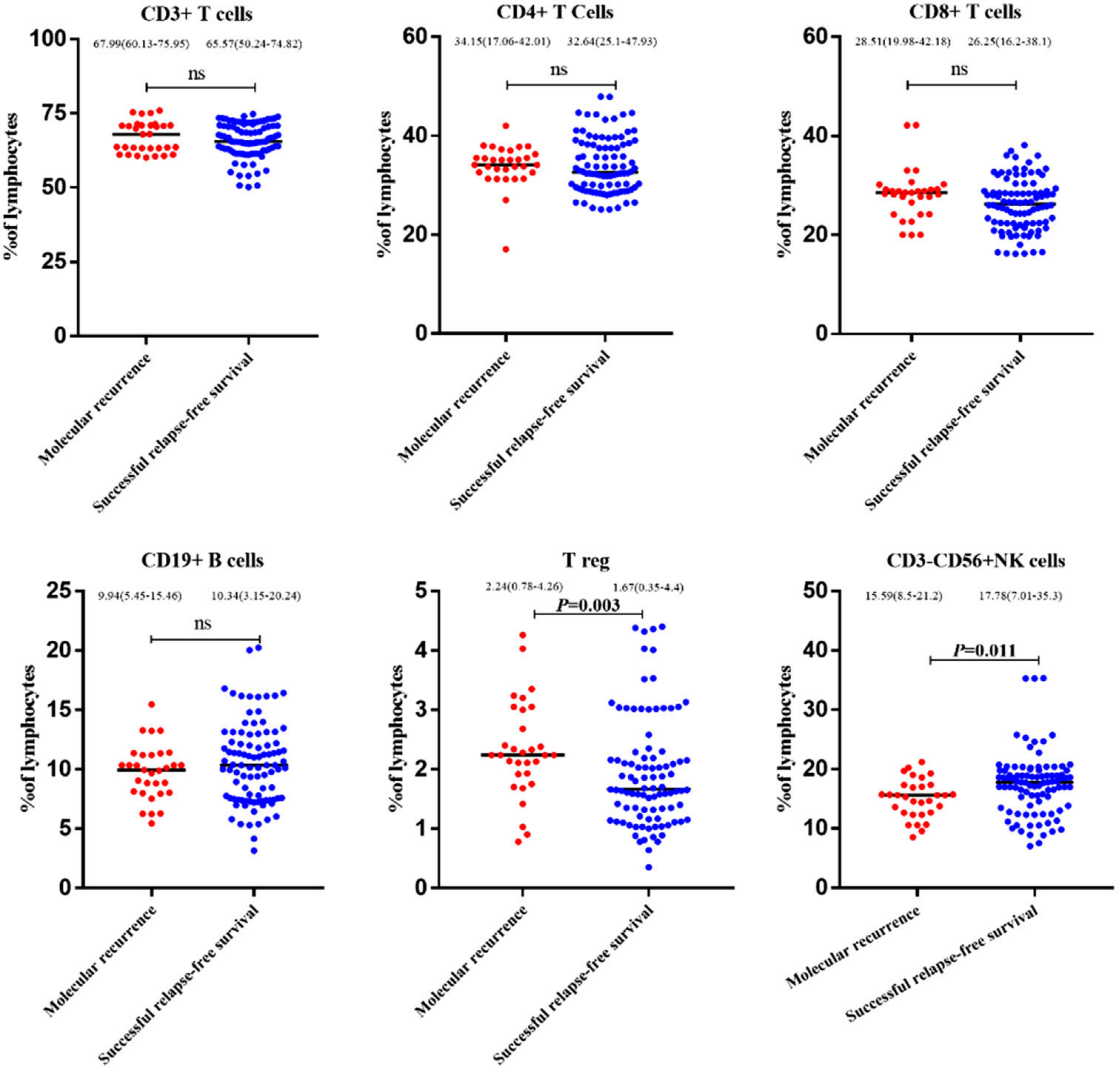
Immunological subsets at the time of de‐escalating or stopping tyrosine kinase inhibitors (TKIs). Bars denote the median. *P*‐values according to the Mann–Whitney *U*‐test are given for each panel, *P*< 0.05 was considered statistically significant, ns means non‐significant

Here, we collected immunological subsets in trial entry and after 6 months of the half‐dose therapy or cessation TKIs. Cytolytic NK cells (CD3^–^CD56^+^CD16^+^) as a proportion of NK cells were significantly increased at 6 months in the de‐escalation group compared with baseline (*P* = 0.048; Table 4). Although the proportion of the other lymphocyte subsets was slightly changed after 6 months of de‐escalation, but the changes were not statistically significant (Table 4). Simultaneously, the percentage of immunological subsets we assessed was somewhat higher at 6 months in the discontinuation group compared with the trial initial, among them, NK cells’ (CD3^–^CD56^+^) proportion was significantly increased after 6 months of TKIs’ cessation (*P* = 0.001; Table 5). To explore the relationship between autoimmune recovery and molecular recurrence, we analyzed the lymphocyte subsets in all patients (29 cases had recurrence and 96 cases had successful relapse‐free survival). We observed that comparing with the relapsed patients, Tregs as a percentage of lymphocytes were decreased (*P* = 0.003, Table 6, Figure 3), and higher proportion of NK cells were occurred in the non‐relapsing group at TKI de‐escalation or discontinuation (*P* = 0.011, 0.007, respectively, Table 6, Figure 3). And no significant association was seen between any other subset and molecular relapse.

**TABLE 4:** Changes of de‐escalation group in lymphocyte subsets compared with baseline (*n* = 62, median, range)

**Subsets**	**Baseline**	**De‐escalation**	***Z* value**	***P* value**
CD3^+^	67.83 (50.70–75.95)	67.15 (56.61–78.96)	−0.145	0.145
CD3^+^CD4^+^	35.83 (28.40–44.67)	36.37 (23.95–41.33)	−1.739	0.082
CD3^+^CD8^+^	26.59 (16.20–38.10)	26.48 (15.32–35.06)	−0.729	0.466
CD19^+^	10.07 (3.15–16.80)	9.47 (4.75–16.75)	−1.658	0.097
CD3^+^CD4^+^CD25^high^CD127^–^Foxp3^+^	1.96 (0.35–4.40)	1.94 (0.37–4.00)	−0.799	0.424
CD3^–^CD56^+^	18.49 (10.66–35.30)	19.03 (8.50–30.37)	−0.063	0.950
CD3^–^CD56^+^CD16^+^	0.59 (0.00–98.08)	0.88 (0.00–94.32)	−1.975	0.048*

* The Wilcoxon matched‐pairs signed ranked test was used to compare variables between de‐escalation group and baseline with a level of significance of 0.05.

**TABLE 5:** Changes of discontinuation group in lymphocyte subsets compared with baseline (*n* = 63, median, range)

**Subsets**	**Baseline**	**Discontinuation**	***Z* value**	***P* value**
CD3^+^	65.00 (50.24–74.00)	65.24 (48.93–74.87)	−1.599	0.110
CD3^+^CD4^+^	31.32 (17.06–47.93)	32.56 (19.27–45.97)	−0.084	0.933
CD3^+^CD8^+^	27.76 (19.98–42.18)	28.22 (16.21–37.40)	−1.451	0.147
CD19^+^	10.34 (5.45–20.24)	10.50 (5.48–18.92)	−1.458	0.145
CD3^+^CD4^+^CD25^high^CD127^–^Foxp3^+^	1.75 (0.78–4.26)	1.75 (0.51–4.32)	−1.085	0.278
CD3^–^CD56^+^	15.31 (7.31–24.68)	15.43 (8.04–41.26)	−3.430	0.001*
CD3^–^CD56^+^CD16^+^	0.84 (0.00–10.02)	1.19 (0.00–14.01)	−0.287	0.774

* The Wilcoxon matched‐pairs signed ranked test was used to compare variables between discontinuation group and baseline with a level of significance of 0.05.

**TABLE 6:** Changes in lymphocyte subsets at TKIs de‐escalation or discontinuation group in MMR and relapsing patients (*n* = 125, median, range)

**Subsets**	**Relapsing (*n* = 29) median (range)**	**MMR (*n* = 96) median (range)**	***Z* value**	***P* value**
CD3^+^	67.99 (60.13–75.95)	65.57 (50.24–74.82)	−0.297	0.766
CD3^+^CD4^+^	34.15 (17.06–42.01)	32.64 (25.10–47.93)	−0.832	0.406
CD3^+^CD8^+^	28.51 (19.98–42.18)	26.25 (16.20–38.10)	−1.921	0.055
CD19^+^	9.94 (5.45–15.46)	10.34 (3.15–20.24)	−0.823	0.410
CD3^+^CD4^+^CD25^high^CD127^–^Foxp3^+^	2.24 (0.78–4.26)	1.67 (0.35–4.40)	−2.985	0.003*
CD3^–^CD56^+^	15.59 (8.50–21.20)	17.78 (7.01–35.30)	−2.550	0.011*
CD3^–^CD56^+^CD16^+^	0.12 (0.00–6.41)	1.33 (0.00–98.08)	−2.688	0.007*

* The Mann–Whitney *U*‐test was used to compare variables from relapsing and non‐relapsing patients with a level of significance of 0.05.

### 3.5 Health‐related quality of life

Table 7 gives the quality of life for each group that is assessed 6 months after enrollment. No significant difference was seen between the two groups regarding the overall health status scores (*P* = 0.889). The functional section of the groups was roughly the same, but the emotional functioning tended to be worse in the discontinuation group (the median scores were 75 vs. 83.33, *P* = 0.038). In terms of symptom scales, fatigue, pain, and pruritus in the de‐escalation group were less frequent (the median scores were 22.22 vs. 33.33, 0 vs. 16.67, 0 vs. 33.33; *P* = 0.024, 0.03, 0.505, respectively). Although the median score of financial difficulties was the same, more patients in the discontinuation set were faced with more severe financial burden [scores ≥ 66.67 in the two groups were 10 patients (15.87%) vs. 5 patients (8.06%), *P* = 0.036]. There were no significant differences between the two groups in the other symptom burden.

**TABLE 7:** EORTC QOL‐C30 scales and other items

	**TKI de‐escalation (*n* = 62) median (range)**	**TKI discontinuation (*n* = 63) median (range)**	*P* value^*^
Global health status			
Global health status	58.33 (25–83.33)	58.33 (25–83.33)	0.889
Functional scales			
Physical functioning	93.33 (40–100)	86.67 (40–93.33)	0.863
Role functioning	83.33 (0–83.33)	83.33 (16.67–83.33)	0.957
Cognitive functioning	83.33 (16.67–100)	83.33 (16.67–100)	0.938
Emotional functioning	83.33 (50–100)	75.00 (25–100)	0.038^*^
Social functioning	66.67 (0–83.33)	66.67 (33.33–83.33)	0.097
Symptom scales/items			
Fatigue	22.22 (0–66.67)	33.33 (0–100)	0.024^*^
Pain	0.00 (0–50)	16.67 (0–100)	0.030^*^
Nausea and vomiting	16.67 (0–66.67)	16.67 (0–100)	0.750
Dyspnea	0.00 (0–33.33)	0.00 (0–33.33)	0.859
Insomnia	33.33 (0–66.67)	33.33 (0–100)	0.936
Appetite loss	33.33 (33.33–100)	33.33 (0–100)	0.441
Constipation	0.00 (0–66.67)	0.00 (0–33.33)	0.673
Diarrhea	0.00 (0–66.67)	0.00 (0–66.67)	0.459
Financial difficulties	33.33 (0–66.67)	33.33 (0–100)	0.036^*^
Pruritus	0.00 (0–100)	33.33 (0–100)	0.505
Edema	0.00 (0–100)	0.00 (0–100)	0.809

*Note*: EORTC QLQ‐C30, European Organization for Research and Treatment of Cancer Quality of Life Questionnaire‐Core 30. The Mann–Whitney *U*‐test was used to compare variables from TKI de‐escalation and discontinuation group with a level of significance of 0.05.

**FIGURE 4:**
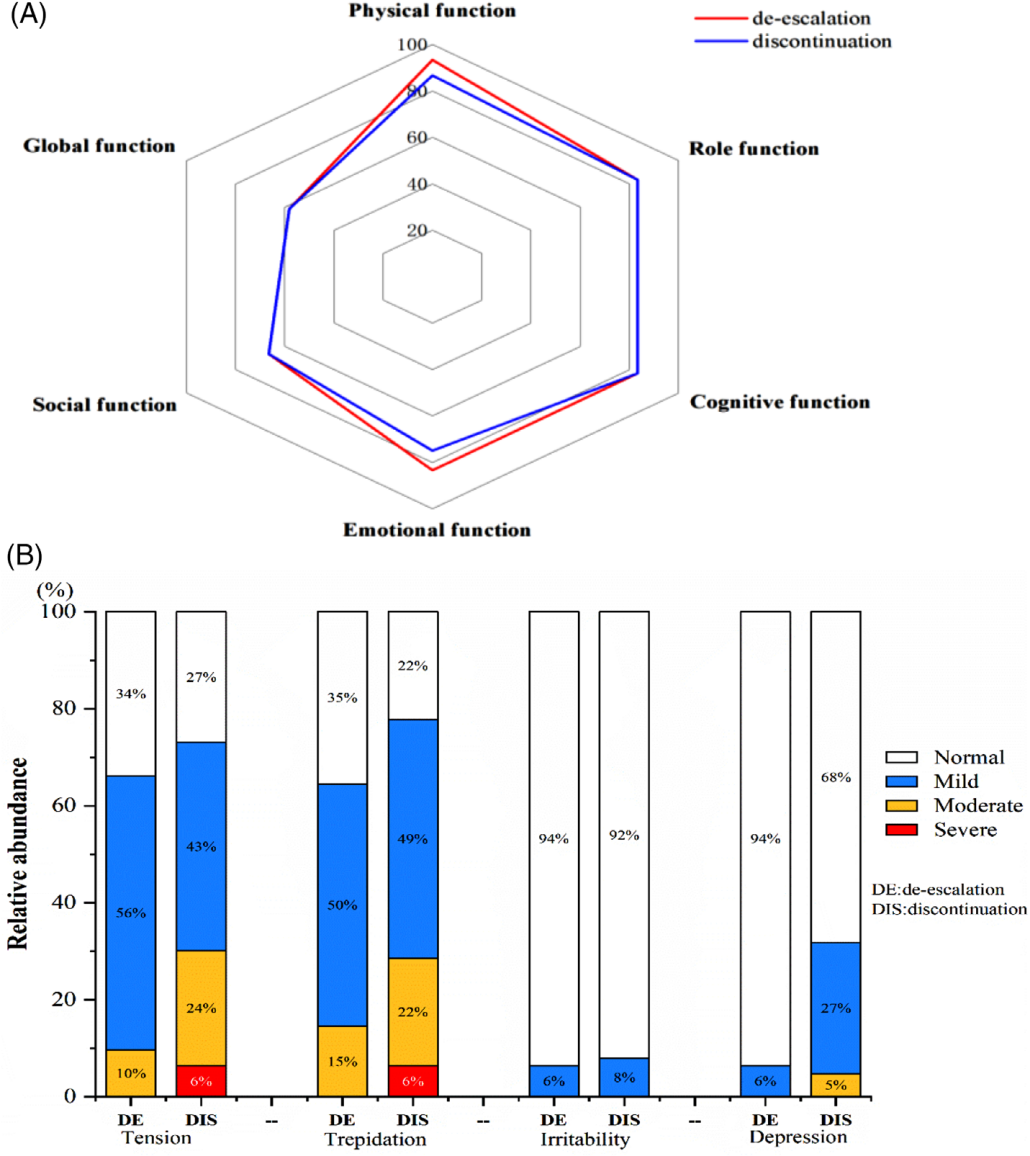
Functional status on de‐escalation vs. discontinuation. (A) A polar plot summarizes scores of six different aspects of function according to patients reported using the EORTC QOL‐C30 scales. As shown, patients on de‐escalation were represented in the outer circle, indicating better function. (B) A histogram shown the relative abundance of four items in the emotional functioning

Next, we analyzed the four items in emotional functioning: tension, trepidation, irritability, and depression, to identify which items bothered the patients most. Figure 4 displays that CML patients showed more tension and trepidation of disease, and people in the discontinuation cohort had higher prevalence and more moderate or severe tension and trepidation of disease than the de‐escalation one [19 patients (30.16%) vs. 6 patients (9.68%);18 patients (28.57%) vs. 9 patients (14.52%), respectively]. When asked the reasons why they feel so nervous and worried about their sickness, the majority of patients complained that molecular recurrence after discontinuing was their biggest concern.

## 4 DISCUSSION

There has been considerable interest in TFR of CML in the past few years, with most studies typically showing recurrence‐free survival (defined as loss of MMR) of 50%–60% in the first chronic phase patients on the TKI therapy for some years and in stable MR4 [4]. The current TKI discontinuation strategies are still too far because of residual LC and leukemia stem cells (LSCs) that cannot be eliminated by patient‐specific immunological mechanisms [5, 16]. Dose optimization of TKIs maybe minimizing side‐effects associated with continuous TKI therapy is required [7]. In our study, 125 patients were enrolled into the de‐escalation group or the discontinuation group, and there were no differences of basic characteristics between two groups. Follow‐up for 12 months, the relapse‐free survival in the de‐escalation set was significantly superior to the discontinuation set [88.32% (95% CI 79–98) vs. 59.98% (95% CI 47–73), *P* = 0.0002]. The proportion of patients who had molecular recurrence (loss of MMR) in the half‐dose group was similar to the de‐escalation phase in DESTINY trial (8.06% vs. 7%) [8], and estimated TFR rate in the discontinuation group was aligned with other prospective studies that had reported data (59.98% vs. 50%–60%) [4]. After 12 months, the frequency of BCR‐ABL1 assessed was maintained at every 3 months. Follow‐up up to now (26 months), as shown in Figure 2A, there were two patients with late relapse in the discontinuation group, who relapsed at months 22 and 25, respectively, and the relapse‐free survival rate decreased to 54.51%. While no new relapse was observed in the de‐escalation group. In our study, 29 patients occurred molecular relapses, all patients returned to MMR within 6 months of TKI resumption. Monitoring BCR‐ABL1^IS^ transcript level frequently is beneficial to detect the loss of MMR timely and re‐administer TKIs early. There were no death or disease progression cases during the 12‐month follow‐up, indicating that reduction or stop of TKIs is feasible and safe for CML‐CP patients who in DMR.

Duration of the treatment before cessation TKIs was reported as a prognostic factor in many studies [9, 17]. In the EUROSKI study [17], the benefit of the additional treatment on recurrence‐free survival is 3% per additional year, this proportion is 4% in the DESTINY trial [8]. In our analysis, the recurrence rate of patients who accepted TKIs for over 6 years was lower than those treated less than 72 months (12/71, 16.90% vs. 19/54, 35.19%). These results displayed that longer the duration of the TKI treatment, the lower the recurrence rate after de‐escalation or discontinuation. We also observed that longer maintaining DMR was predictive for a better outcome, this is consistent with the stop imatinib study (STIM) and the Italy study [18, 19]. Besides, compared with imatinib, the second generation TKIs as first‐line treatment could induce DMR faster, and the second generation TKIs’ first‐line discontinuation trial showed that the relative recurrence risk was reduced to at least 9% [19, 20]. Unfortunately, due to the low usage of the second generation TKIs in our study, this advantage was not presented.

All TKIs have potential immunosuppressive effects [21]. In our study, TKIs cessation was accompanied by a significant increase in CD3−CD56+ NK cells, this is consistent with the inhibitory effect of TKIs on NK‐cell expansion in vitro. And the other lymphocyte subsets also experienced increasing after TKIs discontinuing, as observed by Rea et al. [22]. Alternatively, TKI de‐escalation may modify anti‐leukemic immune responses, in this study, we found that cytolytic NK cells were significantly increased from baseline after 6 months whether in the de‐escalation or TKIs’ cessation group. The immune reconstitution that occurs as a consequence of the TFR, maximal restoration of immune responses occurred only in MR^4.5^, increased NK‐cell and effector‐T cell cytolytic function, reduced T‐cell PD‐1 expression and reduced numbers of monocytic myeloid‐derived suppressor cells (MDSCs) [21]. Some trials also detected that treatment in discontinuation is more successful in patients with lower expression of CD4^+^PD‐1^+^ cells and less successful with higher proportion of MDSCs and CD86^+^ plasmacytoid dendritic cells [10, 13]. Herein, we presented that compared with relapsing patients, the proportion of immunosuppressive cells’ Tregs was decreased and the proportion of CD3^–^CD56^+^ NK cells which can exert detrimental effects to cancer cells was increased in those achieving good clinical results after de‐escalating or discontinuing, these results were consistent with the EUROSKI [17]. Recently, Harrington et al. [23] found that patients with 50 mg dose of dasatinib had significantly higher proportional increase in IL‐2 expression after OKT3 activation in CD4^+^ and CD8^+^ cells compared with patients on 100 mg. Moreover, some stop second generation TKI trials have mentioned that lower CD4^+^ T cells count before discontinuation was a significant favorable prognostic factor for TFR, while a rise in the subset of effector memory CD8^+^ cells predicted molecular relapse [14, 20].

In our study, we failed to find the significant differences of total T cells, CD4^+^ or CD8^+^ T cells between the recurrence and successful TFR groups, illustrating that NK cells‐mediated immune response may play a key role in this study. The contribution of the leukemic stem cell activation and the immune system clearly requires further study and whether increased periods of de‐escalation could mitigate the sharp fall in recurrence‐free survival after subsequent cessation will be interesting to investigate.

The data on the HRQOL of patients during TFR are still limited. Several studies [24, 25, 26] evaluating TFR show stable or improved HRQOL after TKI discontinuation, but 20%−30% of patients manifesting as increased musculoskeletal pain after stopping therapy, little is known about the emotional impact of attempting TFR. Therefore, we used EORTC QLQ‐C30 questionnaire to assess HRQOL in the de‐escalation and discontinuation groups. We found that most functional scales and symptoms were similar in these two groups. But regarding the emotional functioning, TKIs‐off patients tend to be more serious, this may be explained by patients’ fear of relapsing after cessation, reminding us to pay more attention to patients’ mental health when stopping the TKIs. Fatigue and pain consisting of TKIs withdrawal syndrome were significantly worsened in the discontinuation group (*P* = 0.024 and 0.03, respectively), as discovered by Park et al. [25]. And more patients complained newly developed musculoskeletal pain after TKIs’ cessation (*P* = 0.021). Since patients who relapsed would spend more to restart medication and monitor the disease frequently, financial difficulties were more common in the discontinuation group (*P* = 0.036).

In summary, we present data that de‐escalation may improve the proportion of patients in stable MMR for patients who achieved DMR, immune reconstitution maybe one of the mechanisms. Compared with abruptly discontinuation, the de‐escalation group showed better disease‐specific HRQOL. Our results support the rationale for TKI dose de‐escalation in patients who have already reached sustained remission.

## CONFLICT OF INTEREST

The authors declare that there is no conflict of interest that could be perceived as prejudicing the impartiality of the research reported.

## ETHICS STATEMENT

Ethics committee consent was obtained prior to the study.

## PATIENT CONSENT STATEMENT

We have obtained written informed consent or verbal consent from the patient or patient's parent/guardian already.

## Data Availability

The data that support the findings of this study are not publicly available due to privacy or ethical restrictions.
